# Evaluation of Electrical Impedance as a Biomarker of Myostatin Inhibition in Wild Type and Muscular Dystrophy Mice

**DOI:** 10.1371/journal.pone.0140521

**Published:** 2015-10-20

**Authors:** Benjamin Sanchez, Jia Li, Sung Yim, Adam Pacheck, Jeffrey J. Widrick, Seward B. Rutkove

**Affiliations:** 1 Department of Neurology, Division of Neuromuscular Diseases, Beth Israel Deaconess Medical Center, Harvard Medical School, Boston, MA 02215-5491, United States of America; 2 Division of Genetics and Genomics, Boston Children’s Hospital, Harvard Medical School, Boston, MA 02215-5491, United States of America; University of Louisville School of Medicine, UNITED STATES

## Abstract

**Objectives:**

Non-invasive and effort independent biomarkers are needed to better assess the effects of drug therapy on healthy muscle and that affected by muscular dystrophy (mdx). Here we evaluated the use of multi-frequency electrical impedance for this purpose with comparison to force and histological parameters.

**Methods:**

Eight wild-type (wt) and 10 mdx mice were treated weekly with RAP-031 activin type IIB receptor at a dose of 10 mg kg^−1^ twice weekly for 16 weeks; the investigators were blinded to treatment and disease status. At the completion of treatment, impedance measurements, *in situ* force measurements, and histology analyses were performed.

**Results:**

As compared to untreated animals, RAP-031 wt and mdx treated mice had greater body mass (18% and 17%, *p* < 0.001 respectively) and muscle mass (25% *p* < 0.05 and 22% *p* < 0.001, respectively). The Cole impedance parameters in treated wt mice, showed a 24% lower central frequency (*p* < 0.05) and 19% higher resistance ratio (*p* < 0.05); no significant differences were observed in the mdx mice. These differences were consistent with those seen in maximum isometric force, which was greater in the wt animals (*p* < 0.05 at > 70 Hz), but not in the mdx animals. In contrast, maximum force normalized by muscle mass was unchanged in the wt animals and lower in the mdx animals by 21% (*p* < 0.01). Similarly, myofiber size was only non-significantly higher in treated versus untreated animals (8% *p* = 0.44 and 12% *p* = 0.31 for wt and mdx animals, respectively).

**Conclusions:**

Our findings demonstrate electrical impedance of muscle reproduce the functional and histological changes associated with myostatin pathway inhibition and do not reflect differences in muscle size or volume. This technique deserves further study in both animal and human therapeutic trials.

## Introduction

Electrical impedance has been used in a variety of noninvasive physiological monitoring applications including muscle [1–5]. Impedance is normally obtained by measuring the resistive and reactive components at a single or multiple frequencies. In whole body impedance applications, for example, combining single-frequency muscle impedance measures with predictive equations allow estimates of skeletal muscle mass [6], muscle volume [7, 8], fat free mass [9] and total body water [10, 11]. Recently, the use of easily applied, localized impedance measurements of muscle [12] have shown substantial clinical utility. For example, single-frequency muscle impedance measures have shown correlation to clinical parameters in patients with amyotrophic lateral sclerosis [13], muscle injury [14], and in mice with muscular dystrophy [15].

Unlike the single-frequency approach, e.g. impedance at 50 kHz, multi-frequency impedance measures allows the user to obtain a more complete description of the system under investigation [16, 17]. Using models it is possible to summarize multi-frequency information into a reduced set of parameters [18, 19]. The reader can find a comprehensive review on the measurement of impedance and modeling in [20–22] and [23] respectively. Briefly, early models, assumed the addition of circuit elements like resistors and capacitors [24] to account for the dispersion of cells in size and morphology, with cell membrane capacitances and resistances varied across frequency. Later, the introduction of the semi-empirical constant phase element proposed by Cole [25] produced a better model of electrical impedance. Since then, a number of alternatives to the Cole model can be found in the literature [26]. In skeletal muscle, for example, disuse following bone fracture has been analyzed using a five-element circuit model [27]. In cardiac muscle, the authors in [28] used the equivalent circuit model proposed in [29] to study the closure of gap junctions during ischemia.

In this study, we sought to apply the Cole impedance parameters to assess muscle hypertrophy and functional enhancement induced in wild type (wt) and muscular dystrophy (mdx) animals [30] treated with the activin type IIB receptor myostatin inhibitor RAP-031 (Acceleron Pharma, Cambridge, MA, USA). Our interest was to test the ability of impedance to provide a non-invasive, effort-independent biomarker on the condition of muscle and its function. Thus, we compared the impedance data to isometric force recordings and histological indices.

## Materials and Methods

### Animal and drug therapy

All animal procedures were approved by the Institutional Animal Care and Use Committee at the Beth Israel Deaconess Medical Center. Fourteen male wt (C57Bl/6J) and 19 male mdx (C57BL/10ScSn-Dmdmdx/J) mice, obtained from Jackson Laboratories (Bar Harbor, Maine, USA), were treated with RAP-031 or phosphate-buffered saline (PBS) starting at 10 weeks of age. They were assigned to 2 groups: (i), WT_RAP-031_ = 8 and MDX_RAP-031_ = 10 treated with myostatin inhibitor RAP-031 (Acceleron Pharma, Cambridge, Massachusetts, USA); and (ii), WT_untreated_ = 6 and MDX_untreated_ = 9 the untreated groups. Mice were given *ad libitum* access to food (Formulab Diet 5008, LabDiet, St. Louis, Missouri, USA) and water. Untreated mice were injected with PBS, 1× (Corning Cellgro, Manassas, Virginia, USA), and treated mice received RAP-03 at a dose of 10 mg kg^−1^ twice weekly for 16 weeks using 0.5 ml, 0.33 × 12.7 mm insulin syringes (Comfort Point, Los Angeles, California, USA). Syringes were prepared beforehand by a researcher (A.P, S.Y) and were labeled with a number that corresponded to the numerical identity of both the vehicle and RAP-031 treated mice. Then a second researcher (J.L), who was blinded, was given the syringes for injections to reduce any potential bias. Throughout the study, animals were arranged in cages with a maximum of 5 rodents per cage. J.L remained blinded to treatment and disease status throughout the study.

### Impedance system and electrode array

An impedance analyzer (EIM1103, Skulpt Inc., San Francisco, California, USA) was used to measure multi-frequency data at frequencies between 8 kHz and 1 MHz. An electrode array made of four stainless steel strips placed in parallel was used to perform the measurements [31]. The two outer strips were 0.55 mm wide and 3.95 mm long; the two inner strips were 0.55 mm wide and 2.85 mm long. The strips were 0.55 mm apart measured from the center of each strip.

### Force experimental setup

To record the force exerted by the gastrocnemius, we used a high-speed servomotor (model 305C, Aurora Scientific, Aurora, Ontario, Canada). The output force-length signals from the lever system were interfaced to our PC-platform based on a PXI (PCI eXtensions for Instrumentation) system integrating a PXIe-8135 quad-core processor based embedded controller (8 GB DDR3 memory, 1600 MHz), and acquired at a sampling frequency of 1 ks s^−1^ the sampling frequency using a two-channel acquisition board PXI-4461 (204.8 ks s^−1^, 24-bit) from National Instruments (Austin, Texas, USA). A custom program written in LabVIEW (National Instruments) controlled the lever arm movement and the output of a biphasic pulses current muscle stimulator (model 701, Aurora Scientific) using the two analog outputs and one digital output from the board USB-6211 (250 ks s^−1^, 16-bit).

### Animal impedance and force measurements

Impedance and *in situ* force experiments were performed under 1–2% inhaled isoflurane anesthesia delivered by nosecone, with body and muscle temperature being maintained by a heating pad (37°C). After the fur was clipped, a depilatory agent was applied to the skin to remove all remaining fur before the first impedance measurement. Then the skin was cleaned with 0.9% saline solution. The leg was taped to the measuring surface at an approximately 45° extending out from the body. In that position, electrical impedance measurements on the gastrocnemius were performed. Immediately afterward, while still anesthetized, the animal underwent a non-survival surgery in which the gastrocnemius muscle was exposed. The calcaneal tendon was then cut at its insertion point and dissected away from the underlying fascia and soleus muscle. The tendon was then connected to the force lever arm and the leg stabilized by inserting a disposable monopolar needle (902-DMF37-S, Natus neurology, Middleton, Wisconsin, USA) through the knee joint [32].

Twitch force was recorded after stimulation by a single stimulus using 200 *μ*s square pulse delivered to insulated electrocardiogram needles (F-E2M-48, Grass Technologies, Warwick, Rhode Island, USA) stimulating the sciatic nerve at the sciatic notch. Stimulation current and muscle length were adjusted to maximize tetanic force during a 200 ms duration stimulus train at 120 Hz. Before isometric force data collection, the optimal length *L*_*o*_ was measured with digital calipers as the distance between the knee and the calcaneal tendon. All subsequent isometric data were collected at this pulse duration, stimulation current, and muscle length. Tetanic force frequency relationship was recorded after stimulation by a square wave stimuli of 200 ms.

At the conclusion of all studies, the animals were sacrificed via the use of a stream of carbon dioxide.

### Histology

Excised gastrocnemius muscle tissue was flash frozen in isopentane, which had been cooled by liquid nitrogen, and was stored at -80°C. Each tissue corresponding to the largest part of the muscle was cut into 5 *μ*m slices, placed on glass slides, and stained with hematoxylin and eosin. Utilizing a computer (Dell Optixflex 380) and Zeiss Axiophot microscope with a motorized stage, a total of WT_untreated_ + WT_RAP-031_ = 13 and MDX_untreated_ + MDX_RAP-031_ = 18 muscle sample tissues were selected, and *N* = 100 myofibers and size were counted and measured for each tissue (3100 myofiber in total, one wt and one mdx mouse were not determined). The evaluator (S.Y) made a non-biased quantification of myofiber size using Stereo Investigator software (MBF Biosciences Inc., Williston, Vermont, USA) and was blinded from the muscle phenotype and therapy. An estimate of the myofiber area was obtained by manually delimiting each single myofiber contour and approximating the shape by an octagon. There was no attemps to discriminate between muscle myofiber types in the quantification.

## Analysis

All calculations were performed using Matlab (The MathWorks, Natick, Massachusetts, USA).

### Isometric force contraction during tetanic stimulation

Tetanic peak force was determined as the peak active force measured at the time when the derivative of the force was 0. The force-frequency relationship was described by the following sigmoid function

F(f;θ)=Fmin+Fmax-Fmin1+(Kf)s, (1)

where *f* is the stimulus frequency and *θ* represents the vector parameters *θ* = [F_min_ F_max_ *K* *s*]; F_min_ is the minimum force, F_max_ is the maximum force, *K* is the inflection point of the curve and *s* is the slope of the sigmoid.

### Electrical impedance

Mean impedance and the sample standard deviation were calculated in transverse direction. The mean impedance was fit to the complex impedance model described by Cole in [25],

Z(jω;Φ)=R∞+R0-R∞1+(jωωc)α, (2)

using as weights the inverse of the sample standard deviation as described in the Appendix, where *ω* is the (angular) frequency in rad s^−1^; Φ = [*R*_0_ *R*_∞_ *ω*_*c*_ *α*] is the Cole vector parameters; and $j=\sqrt{-1}$ is the imaginary number. The central (angular) frequency *ω*_*c*_ corresponds to the frequency with the highest absolute value of the imaginary part of the impedance. The *α* parameter explains the dispersion in the fiber membrane capacitances measured and is related to the dispersion of the shape and size distribution. The case when *α* = 1 is the ideal case when fibers are perfectly homogenous as proposed by Fricke and Morse in [24]. The resistances *R*_0_ and *R*_∞_ model the resistances when *ω* → 0 and *ω* → ∞ respectively.

### Statistics

Mann-Whitney U-test (two-tailed) was used to analyze data for comparisons between untreated and treated groups in wt and mdx mice respectively. The statistical significance was set at *p* < 0.05.

For the wt and mdx mice histology data, the estimation of a normal and inverse Gaussian probability density function parameters and the standard errors were obtained using a maximum likelihood estimator [33].

## Results

### RAP-031 treatment increases body mass and muscle mass

**Fig 1:**
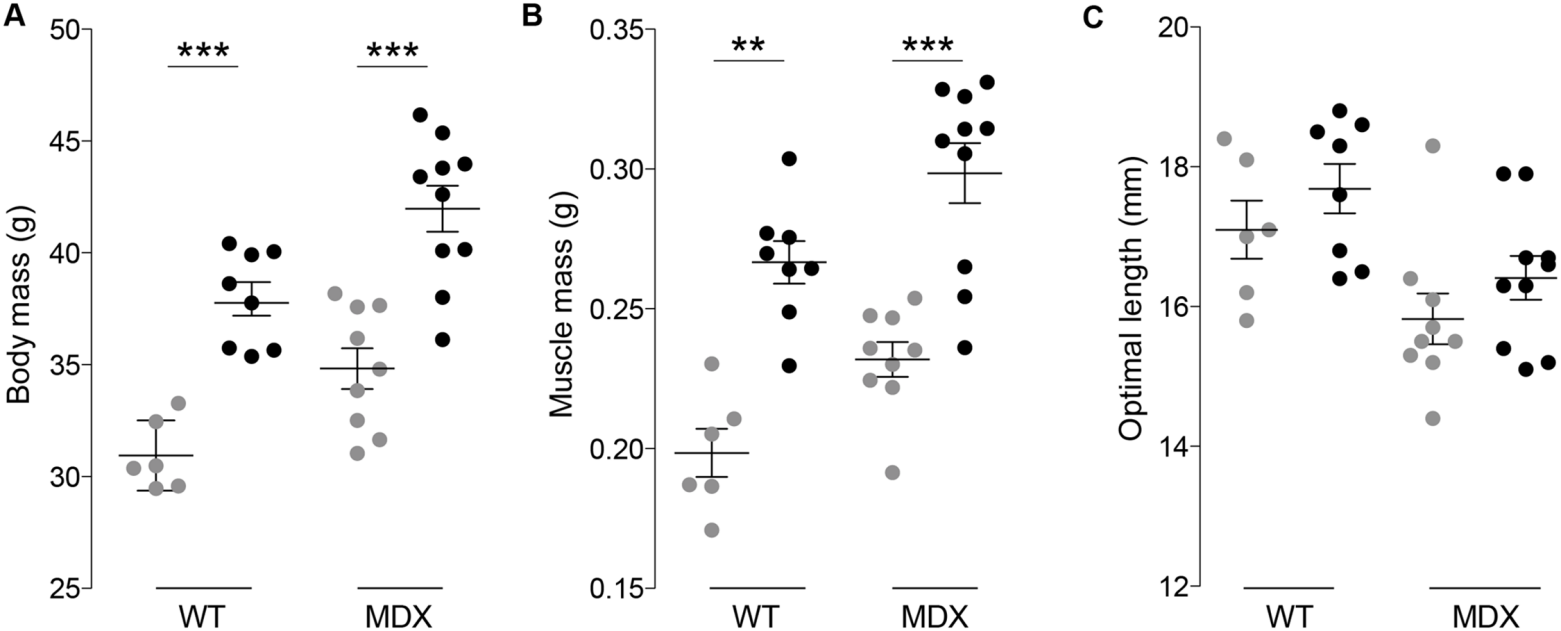
Wild-type (wt) and muscular distrophy (mdx) mice body mass (A); muscle mass (B); and optimal length (C, S1 Table). The horizontal bars are the mean and standard error of the mean. Colors: gray dots, untreated; black dots, RAP−031. ** *p* < 0.01, *** *p* < 0.001.

**Fig 2:**
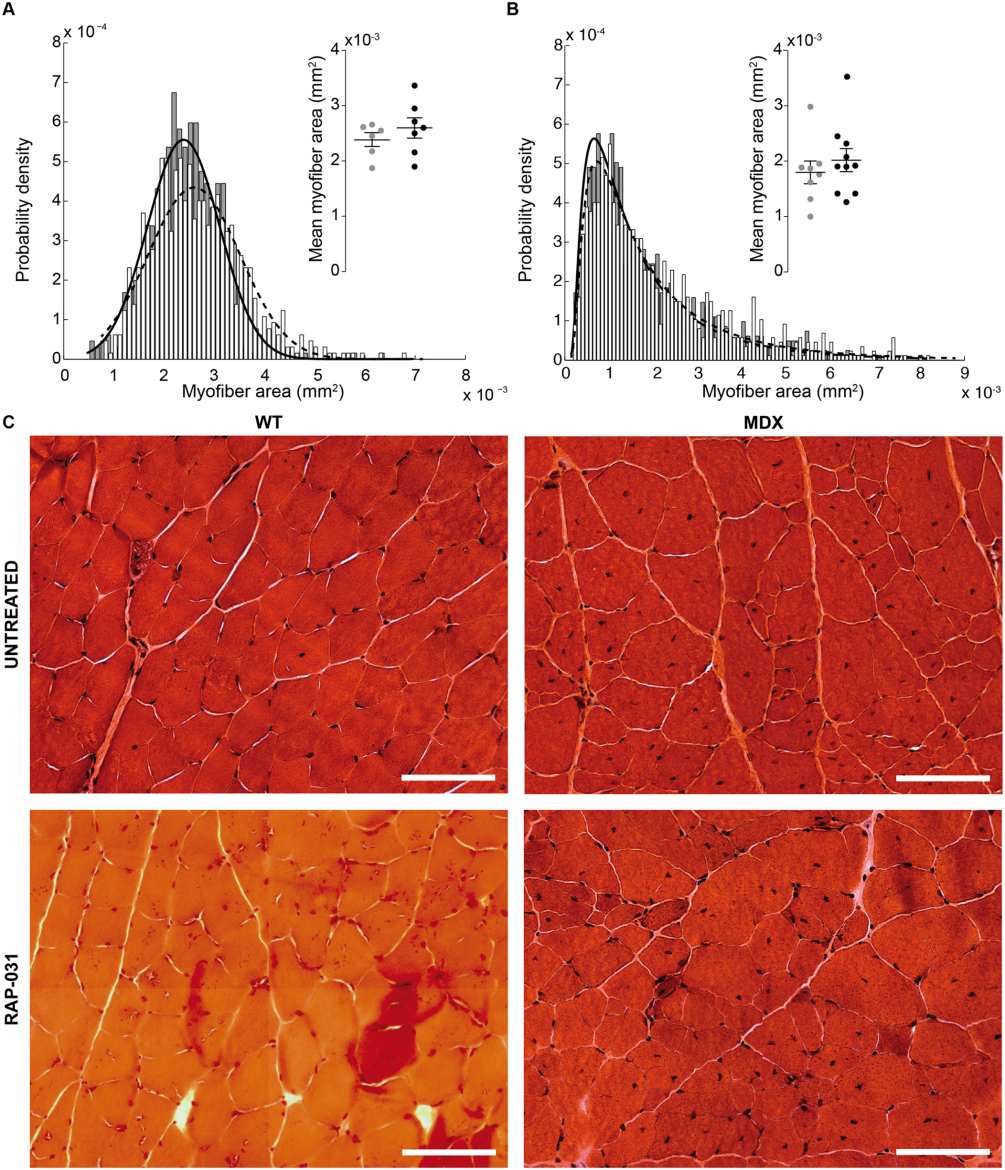
Wild-type (wt, A) and muscular distrophy (mdx, B) myofiber cross-sectional area distribution plot (1300 and 1800 myofibers respectively). WT normal distribution mean $\hat{\mu }$ and spread $\hat{s}$ parameters: ${\hat{\mu }}_{\text{WT, untreated}}\pm {\hat{\sigma }}_{{\hat{\mu }}_{\text{WT, untreated}}}=2387\pm 29$ *μ*m^2^, ${\hat{s}}_{\text{WT, untreated}}\pm {\hat{\sigma }}_{{\hat{s}}_{\text{WT, untreated}}}=719\pm 21$ *μ*m^2^, ${\hat{\mu }}_{\text{WT, RAP-031}}\pm {\hat{\sigma }}_{{\hat{\mu }}_{\text{WT, RAP-031}}}=2597\pm 35$ *μ*m^2^, ${\hat{s}}_{\text{WT, RAP-031}}\pm {\hat{\sigma }}_{{\hat{s}}_{\text{WT, RAP-031}}}=918\pm 25$ *μ*m^2^. MDX inverse normal distribution mean *μ* and shape *λ* parameters: ${\hat{\mu }}_{\text{MDX, untreated}}\pm {\hat{\sigma }}_{{\hat{\mu }}_{\text{MDX, untreated}}}=1795\pm 56$ *μ*m^2^, ${\hat{\lambda }}_{\text{MDX, untreated}}\pm {\hat{\sigma }}_{{\hat{\lambda }}_{\text{MDX, untreated}}}=2273\pm 114$ *μ*m^2^, ${\hat{\mu }}_{\text{MDX, RAP-031}}\pm {\hat{\sigma }}_{{\hat{\mu }}_{\text{MDX, RAP-031}}}=2018\pm 58$ *μ*m^2^, ${\hat{\lambda }}_{\text{MDX, RAP-031}}\pm {\hat{\sigma }}_{{\hat{\lambda }}_{\text{MDX, RAP-031}}}=2460\pm 110$ *μ*m^2^. In detail, the distribution of the estimated mean values ${\hat{\mu }}_{\text{WT}}$ and ${\hat{\mu }}_{\text{MDX}}$ (see S1 Table). The horizontal bars are the mean and standard error of the mean. Colors: gray dots, solid black line and gray bars, untreated; black dots, dashed black line and white bars, RAP−031. (C) Hematoxylin and eosin staining of gastrocnemius. The scale bar indicates 100 *μ*m (40X magnification).

As expected from results of others [34–36], blocking the myostatin pathway led to an increase in body mass and muscle mass. Specifically, the administration of the compound in wt and mdx mice was associated, respectively, with a greater body mass of 18% and 17% (*p* < 0.001, Fig 1A), muscle mass of 25% and 22% (*p* < 0.01 and *p* < 0.001 respectively, Fig 1B) and non-significant increase of optimal length of 3.3% and 3.6% (*p* = 0.28 and *p* = 0.25 respectively, Fig 1C). The gross muscle alterations were mirrored by a limited rightward shift in the myofiber size (Fig 2). Specifically, the mean myofiber size was 8% and 12% greater in treated treated wt and mdx mice versus untreated animals. However, these changes were found not significant with *p* = 0.44 and *p* = 0.31 respectively. These changes in myofiber size were more modest than those found in other studies and is discussed in more detail below.

### The half relaxation time in response to a single twitch was reduced in treated mdx animals

**Fig 3:**
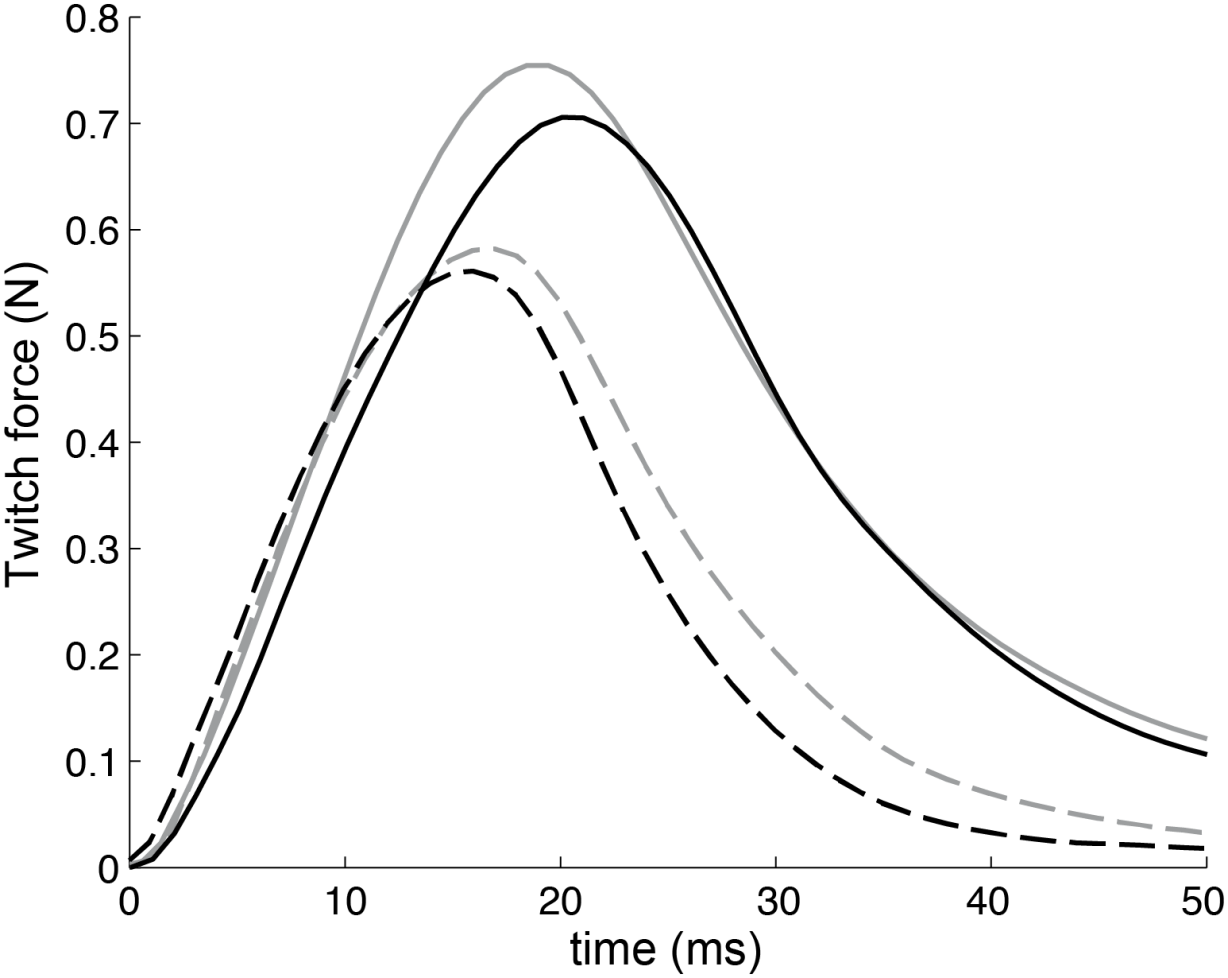
Examples of the qualitative differences in the time courses of isometric twitches induced by the treatment (data in Table 1). *In situ* contraction force of the gastrocnemius muscle. Colors: gray, untreated; black, RAP-031. Groups: solid line, wild-type mice; dashed line, muscular dystrophy mice.

The maximum twitch force (Fig 3, Table 1) generated by gastrocnemius muscles in response to treatment was non-significantly greater in both wt and mdx animals than untreated animals (13% *p* = 0.16 and 10% *p* = 0.19, respectively). Interestingly, treated mdx mice had a trend toward faster relaxation compared to untreated (15%, *p* = 0.07). This reduction in the half-relaxation time was consistent with a border-line 15% increase (*p* = 0.06) in the minimum of the force derivative.

**Table 1:** Twitch measures. Mean and standard error of the mean.

	Contraction time (ms)	Maximum force (N)	Half relaxation time (ms)	Maximum slope contraction (mN s^−1^)	Minimum slope relaxation (mN s^−1^)
WT_untreated_	20.2±0.8	0.65±0.03	13.2±1	49.9±2.0	-37.6±4.1
WT_RAP-031_	19.0±1.7	0.75±0.05	12.3±0.5	59.5±5.0	-43.4±2.5
*p*	0.49	0.16	0.41	0.14	0.22
MDX_untreated_	15.9±0.88	0.45±0.03	11.4±0.89	48.8±2.6	-31.6±1.7
MDX_RAP-031_	16.4±0.20	0.50±0.02	9.6±0.35	51.4±2.8	-37.1±2.1
*p*	0.55	0.19	0.07	0.52	0.06

### RAP-031 treatment increases maximum isometric force of the gastrocnemius muscle in wt mice but not in mdx animals

**Fig 4:**
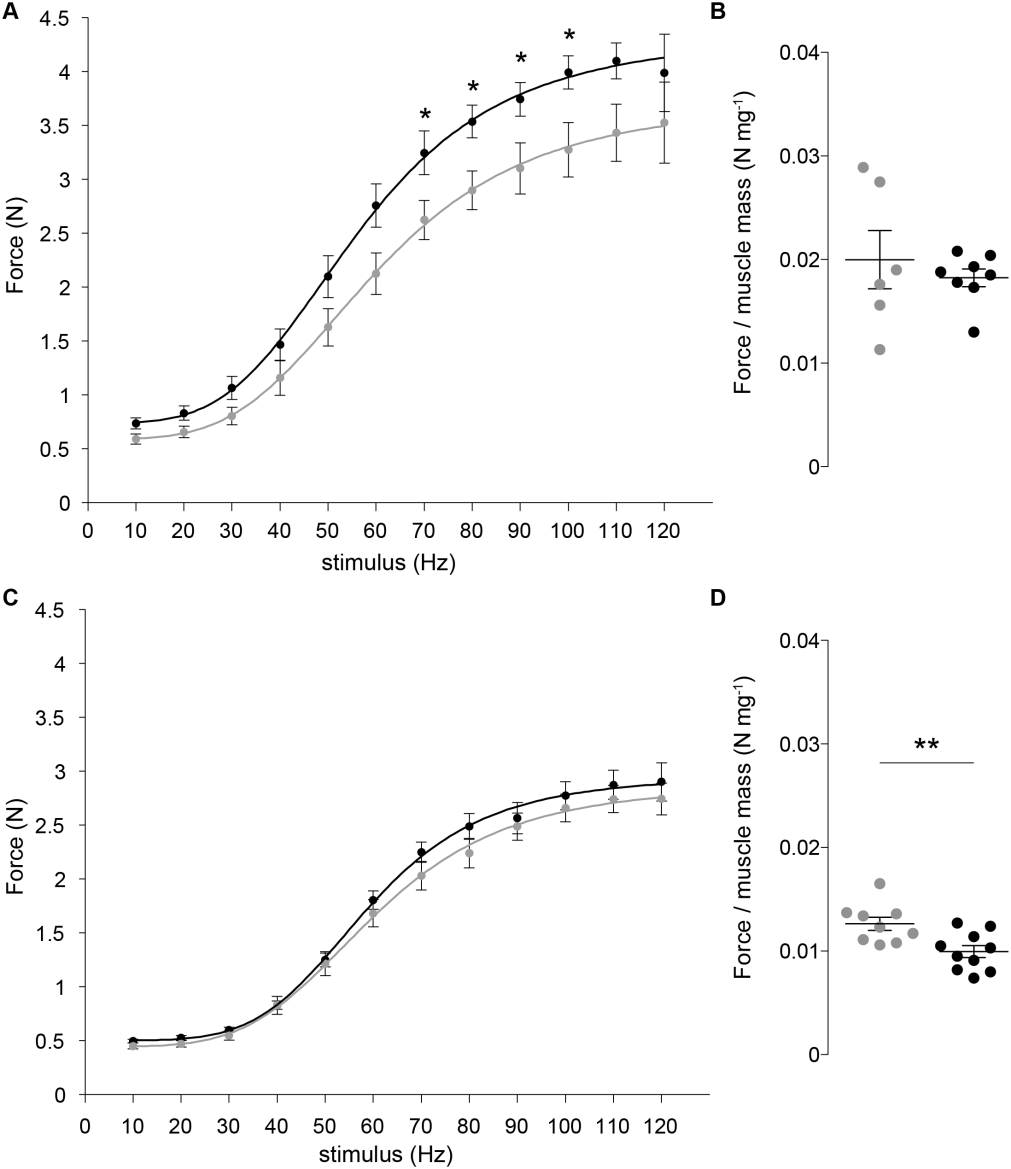
Wild-type (wt) (A) and muscular dystrophy (mdx) mice (C) force-frequency relationship. The dots and the error bars are, respectively, the mean maximum isometric force and standard deviation. The lines are the sigmoid model Eq (1). WT (B) and mdx mice (D) maximum force per muscle mass respectively (see S1 Table). The horizontal bars in (B, D) denote the mean and standard error of the mean. Colors: gray, untreated; black, RAP-031. * *p* < 0.05, ** *p* < 0.01.

The maximum isometric force generated by gastrocnemius muscle in wt animals in response to treatment was higher than in untreated (*p* < 0.05) at stimulation frequencies from 70 to 100 Hz (Fig 4A). *In situ* force reported in [37] of the entire triceps surae (the gastrocnemius comprises 85% of the mass of the triceps surae muscle group) as 3.7 N is in good agreement with our value of 4 N. In order to assure that this agreement is not due to differences in the size of muscles by Ashton-Miller and ourselves, we divided *in situ* force by muscle mass, obtaining a value of 0.016 N mg^−1^ [37]. This is on very good agreement with our value of 0.020 N mg^−1^ for the untreated wt mice (Fig 4B). When force was normalized to muscle mass; however, it did not differ from the untreated mice, a finding consistent with work by others in specific force [34]. The lack of a difference in the wt treated and untreated groups suggests the treatment did not alter the fundamental mechanisms underlying contraction as the increase in maximum force was proportional to the increase in muscle mass. Therefore, RAP-031’s effect in wt mice appears to be quantitative rather than qualitative. In treated mdx mice, the increase in mean myofiber size did not translate into an effective net change in active force (Fig 4C) resulting in an 21% (*p* < 0.01) lower force per unit of muscle mass (Fig 4D).

### Multi-frequency impedance parameters detect muscle structural changes induced by RAP-031 treatment

**Fig 5:**
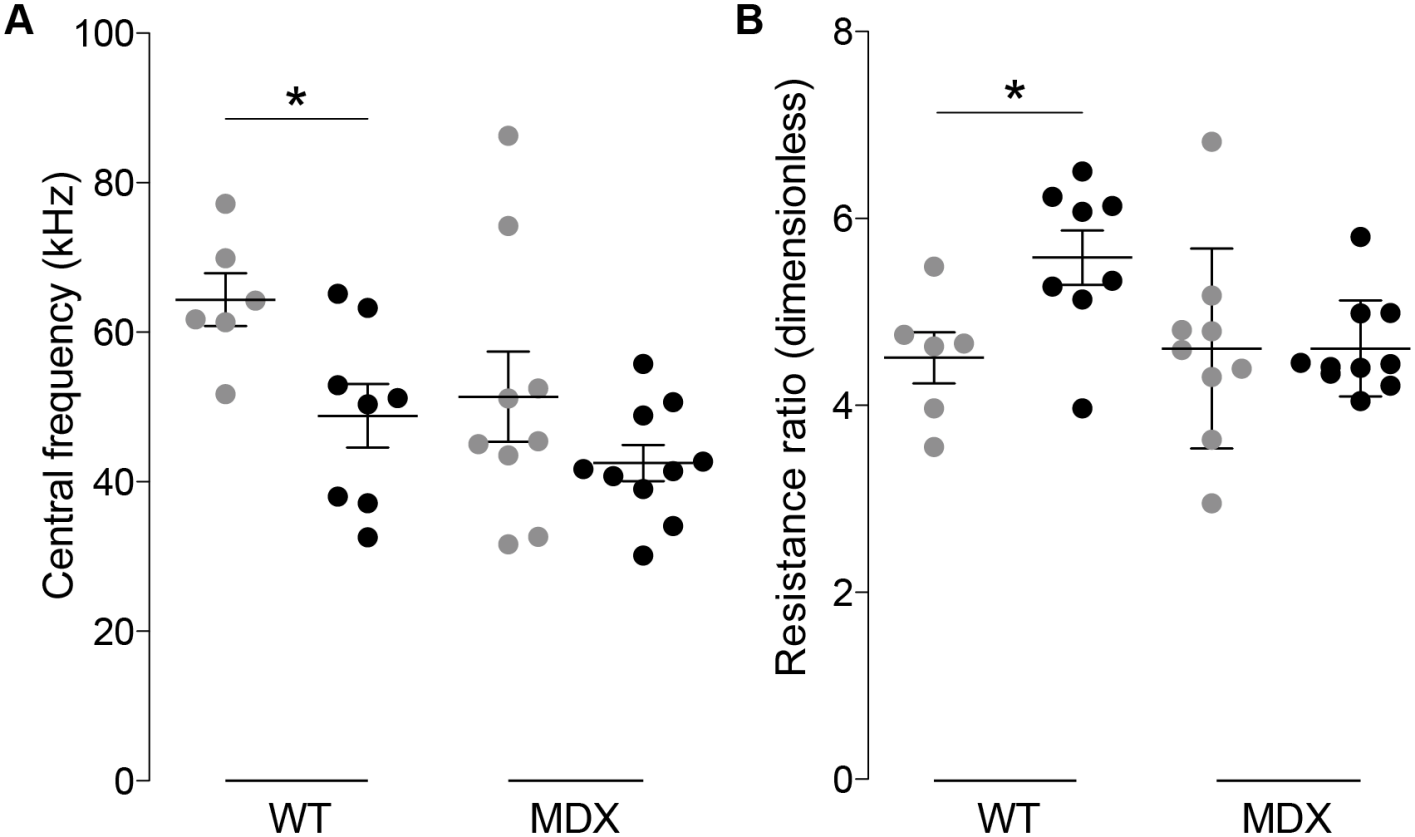
Wild-type (wt) and muscular dystrophy (mdx) Cole impedance parameters: central frequency ${\hat{\omega }}_{c}/2\pi$ (kHz) and resistance ratio ${\hat{R}}_{0}/{\hat{R}}_{\infty }$ (dimensionless). The estimated parameters and their standard errors are shown in S1 Table.

RAP-031 was administered to mice in order to determine if blocking myostatin was detectable in the muscle impedance. Despite the relatively modest effects on isometric force and changes in myofiber histology, the electrical impedance parameters did detect significant differences and, in general, corresponded to the observed changes. Of most interest, the central frequency showed treated wt mice had a 24% (*p* < 0.05) lower central frequency, although the difference in mdx animals was non-significant (17%, *p* = 0.21) (Fig 5A). Similarly, the 19% (*p* < 0.05) higher resistance ratio in the wt mice helps confirm the reduction in extracellular space accompanying the larger fibers in the wt animals (Fig 5B). The lack of analogous differences in the mdx animals suggest that, despite the potentially slightly greater area of the myofibers, other pathological alterations associated with the disease (e.g. atrophy) may be occurring such that effect of treatment could not be detected with impedance.

## Discussion

Even though the effects of treatment with RAP-031 were more modest than anticipated, the results of this fully blinded study showed that the combination of multi-frequency electrical impedance with a reduced set of model parameters allowed us to detect the histological changes associated with myostatin inhibition in wt mice, with consistent if not significant effects observed in mdx mice.

We based our analysis on the Cole theory [25, 26] to explain the relationship between the multi-frequency impedance data and structural properties of muscle [1, 38] and not through a randomly correlation analysis. In particular, we found the central frequency did detect alterations in the structure of skeletal muscle induced by the administration of RAP-031 in wt and mdx mice, although only reaching statistical significance in the wt animals. The decrease in the central frequency, a parameter that is inversely related to cell size [39], in treated mice is consistent with myofiber hypertrophy. Furthermore, the increase of the Cole resistance ratio in wt mice, a known estimator of cell density [40, 41], is in good agreement with the increase in mean myofiber size observed. Given a certain muscle volume measured, if the myofiber density increases, the resistance at 0 Hz, as modeled by *R*_0_, increases due a reduction in the extracellular space. The normalization with respect to *R*_∞_ cancels out potential artifacts that may lead to changes in the measured impedance. In mdx mice, however, the Cole resistance ratio was found not to be a sensitive to detect this therapy effect, possibly due to the presence of myofiber atrophy (the median of the untreated wt and mdx distributions shown in Fig 2A and 2B is 2.4⋅10^−3^ mm^2^ and 0.6⋅10^−3^ mm^2^ respectively). Finally, these findings serve to support the fact that localized muscle impedance measurements with the appropriate electrode configuration [42] measure the structural properties of muscle and not muscle volume or size. If impedance was dependent on muscle size more than its compositional and architectural properties, we would have expected to observe a much greater differences in the Cole parameters in both wt and mdx animals.

The non-significantly larger myofiber size in both treated mdx and wt animals was similar to the 11% increase observed in extensor digitorum longus (EDL) at 60 mg kg^−1^ dose reported in [34] but far less than the 29% in the soleus at 10 mg kg^−1^ in [35]. Or the approximately 60% increase in myofiber size in EDL shown in [36] at 10 mg kg^−1^. Similarly, several studies have reported that inhibition of activin-signaling in mdx mice improves the active force of the EDL and either improves or maintains the specific force of this muscle [36, 43, 44]. On the other hand, these beneficial effects of activin signaling inhibition appear to be muscle specific. Both the mdx soleus and diaphragm show little improvement in force with this type of treatment, and in some cases, a reduction in function has been reported [43, 44]. Thus, taken together these effects of activin signaling inhibition appear to be very variable in terms of the muscle being studied; it may simple be that gastrocnemius responds less to the drug than EDL or soleus.

There are several possibilities that can account for this discrepancy in the hypertrophy effect observed as that reported by others. First, the authors in [34] and [35] administered the myostatin inhibitor for a shorter period of time (4 weeks) than we did (16 weeks). In the one study we identified in which the drug was given for a longer period of time 12 weeks (at 1 mg kg^−1^ and 10 mg kg^−1^ doses) and in which EDL force was measured [36], only the lower dose produced an increase in specific force.

In addition to length of treatment, muscle use could also play a role, as the compound has a greater effect on type I fibers than type II fibers [35]. In the present study, animals were housed approximately 4–5/cage, which has been associated with decreased mobility which could have resulted in decreased activity and a reduced drug effect than if the animals were singly housed (other studies evaluating the myostatin pathway did not record mouse housing characteristics to our knowledge).

In the present study, mdx mice were treated with the same high dose as Pistilli *et al* [36]. Although they identified significant increases in single twitch force as compared to the changes observed here, they did not note any increase in the speed of relaxation as our data suggested. Such increased relaxation speed could be secondary to drug-induced changes in the ion channels/sodium-calcium exchanger at plasma membrane, modified the sarco/endoplasmic reticulum isoform/content or altered the compliance/stiffness in sarcomeres and extracellular cellular membrane.

The finding of little improvement in mdx treated isometric force is consistent with the idea that long-term, high dose RAP-031 treatment may not be beneficial to specific force in mdx mice, which could explain the 21% (*p* < 0.01) drop in maximum force normalized by muscle mass in mdx mice. Recent studies show that in the absence of myostatin, which signals via activin IIB receptor, there is a reduction in atrogin-1 levels [45]. Because atrogin-1 is a key enzyme involved in the ubiquitination of proteins for their eventual degradation by the proteasome [46], it has been proposed that life-long disruption of normal myostatin signaling leads to an accumulation of damaged contractile proteins that do not function properly, leading to some degree of contractile dysfunction [45]. Whether the loss of force per muscle mass here, with shorter-term inhibition of the entire activin-signaling pathway is caused by a similar mechanism awaits additional research.

In summary, localized multi-frequency impedance measurements of muscle with Cole analysis can be useful for identifying the effects of the drug therapy and providing a non-invasive, effort-independent approach to assessing the functional properties of muscle. Accordingly, additional studies employing this approach in the study of therapy in neuromuscular disease are warranted.

## Appendix: force and impedance data fitting

The force-frequency relationship and the impedance data were fit to their corresponding model using a Marquardt-Levenberg nonlinear least square curve-fitting algorithm [47]. The maximum number of iterations and the fitting tolerance were set to 10^6^ and 10^−12^ respectively. From the optimal curve-fit parameters, the Jacobian matrix **J**_*n*×*n*_*θ*__ was calculated numerically as

$$\begin{array}{c} J_{ij}={\frac{\partial G(x_{i};\Theta_{j})}{\partial \Theta_{j}}|}_{\Theta_{j}=\Theta_{j}^{\text{opt}}}, \end{array}$$ (3)

with G = {Z, F} the models in Eqs (2) and (1); Θ ∈ {Ω, Φ} the set of vectors parameters; *n* = {12, 37} the number of observations of the independent variable *x* = {*f*, *ω*}; and *n*_*θ*_ = {4, 4} the number of model parameters in Θ. The covariance matrix was estimated from the Jacobian using a diagonal matrix **W** containing as weights the inverse of the data standard deviation,

$$\begin{array}{c} {\text{cov}}_{\Theta }\approx {\left(2\text{Real}\{J^{H}\text{WJ}\}\right)}^{-1}, \end{array}$$ (4)

where the superscript ^*H*^ denotes the hermitian (conjugate transpose) operator. The asymptotic standard deviation for the optimal model parameters was finally obtained from the diagonal elements of the covariance matrix,

$$\begin{array}{c} {\hat{\sigma }}_{\hat{\Theta }}=\sqrt{\text{diag}\{{\text{cov}}_{\Theta }\}}. \end{array}$$ (5)

## Supporting Information

S1 TableEstimated parameters $\hat{x}$ and their standard errors ${\hat{\sigma }}_{\hat{x}}$ in wild-type (wt) and muscular dystrophy (mdx) mice.Parameters: *μ* and *s*, mean myofiber area and standard deviation; *ω*_*c*_, Cole central frequency; *R*_0_/*R*_∞_ Cole resistance ratio; F, maximum isometric force; *m*, muscle mass; *L*_*o*_, optimal length.(PDF)
