# Supplementary material for: Evaluation of Electrical Impedance as a Biomarker of Myostatin Inhibition in Wild Type and Muscular Dystrophy Mice
Source: PLoS One. 2015 Oct 20;10(10):e0140521. doi: 10.1371/journal.pone.0140521 (PMC4618134; doi:10.1371/journal.pone.0140521)
Supplement: S1 Table — Parameters: μ and s, mean myofiber area and standard deviation; ω c, Cole central frequency; R 0/R ∞ Cole resistance ratio; F, maximum isometric force; m, muscle mass; L o, optimal length. (PDF) [file pone.0140521.s001.pdf]

Supplementary materials for

Evaluation of electrical impedance as a biomarker of myostatin inhibition in wild type and muscular dystrophy mice

Benjamin Sanchez[1,*], Jia Li[1], Sung Yim[1], Adam Pacheck[1], Jeffrey J Widrick[2], Seward B Rutkove[1]

1 Department of Neurology, Division of Neuromuscular Diseases, Beth Israel Deaconess Medical Center, Harvard Medical School, Boston, MA 02215-5491, USA.
2 Division of Genetics and Genomics, Boston Children's Hospital, Harvard Medical School, Boston, MA 02215-5491, USA.

*** Corresponding author**
E-mail: bsanchez@bidmc.harvard.edu

Supplementary Table 1. Estimated parameters $\widehat{x}$ and their standard errors $\widehat{\sigma}_{\widehat{x}}$ in wild-type (wt) and muscular dystrophy (mdx) mice. Parameters: μ and s, mean myofiber area and standard deviation; ω_c, Cole central frequency; R_0/R_∞ Cole resistance ratio; $\mathcal{F}$, maximum isometric force; m, muscle mass; L_o, optimal length.

	Animal	$\widehat{\mu} \pm \widehat{\sigma}_{\widehat{\mu}}$† ($\mu$m^2)	$\widehat{s} \pm \widehat{\sigma}_{\widehat{s}}$† ($\mu$m^2)	$\widehat{\omega}_c \pm \widehat{\sigma}_{\widehat{\omega}_c}$ (kHz)	$\widehat{R}_0/\widehat{R}_\infty \pm \widehat{\sigma}_{\widehat{R}_0/\widehat{R}_\infty}$§ -	$\widehat{\mathcal{F}}_{\max} \pm \widehat{\sigma}_{\widehat{\mathcal{F}}_{\max}}$ (N)	$m^\star$ (g)	$L_o^\star$ (mm)
$\mathrm{WT}_{\mathrm{untreated}}$	1	2614±85	848±60	61.4±1.4	5.5±0.09	3.7±0.1	0.21	17.1
	2	2175±45	454±32	61.7±0.7	4.6±0.04	4.7±0.2	0.17	18.1
	3	1871±66	656±47	64.2±1.9	4.7±0.08	5.4±0.3	0.19	16.2
	4	2659±52	523±37	69.9±0.7	3.6±0.02	2.6±0.1	0.23	18.4
	5	2454±82	816±58	77.1±1.3	4.8±0.08	2.9±0.1	0.19	15.8
	6	2548±60	599±43	51.7±0.2	4.0±0.03	3.9±0.1	0.21	17.0
$\mathrm{WT}_{\mathrm{RAP-031}}$	1	2601±98	984±70	32.6±2.0	6.1±0.14	3.5±0.1	0.27	16.5
	2	2713±87	869±62	37.2±0.5	6.2±0.06	4.7±0.1	0.23	16.4
	3	3364±89	893±64	50.4±1.1	5.3±0.07	5.1±0.3	0.26	18.8
	4	2155±96	957±68	52.9±0.7	5.3±0.05	5.2±0.2	0.28	17.6
	5	2949±69	694±49	38.0±1.1	6.1±0.11	4.9±0.1	0.28	18.6
	6	2500±57	571±41	63.3±0.6	6.5±0.15	5.2±0.2	0.25	18.3
	7	*	*	65.1±1.2	3.4±0.06	5.6±0.6	0.30	18.5
	8	1898±53	533±38	51.2±2.0	5.1±0.12	4.6±0.1	0.26	16.8

	Animal	$\widehat{\mu} \pm \widehat{\sigma}_{\widehat{\mu}}$‡ ($\mu$m^2)	$\widehat{\lambda} \pm \widehat{\sigma}_{\widehat{\lambda}}$‡ ($\mu$m^2)	$\widehat{\omega}_c \pm \widehat{\sigma}_{\widehat{\omega}_c}$ (kHz)	$\widehat{R}_0/\widehat{R}_\infty \pm \widehat{\sigma}_{\widehat{R}_0/\widehat{R}_\infty}$§ -	$\widehat{\mathcal{F}}_{\max} \pm \widehat{\sigma}_{\widehat{\mathcal{F}}_{\max}}$ (N)	$m^\star$ (g)	$L_o^\star$ (mm)
$\mathrm{MDX}_{\mathrm{untreated}}$	1	1317±119	1602±227	52.6±0.4	4.8±0.006	2.7±0.4	0.25	16.4
	2	1760±100	5420±766	32.8±0.3	4.4±0.003	3.7±0.2	0.22	15.3
	3	1618±180	1297±183	45.6±0.3	4.6±0.003	3.4±0.2	0.25	15.5
	4	2982±195	6967±985	51.3±2.2	4.3±0.008	2.6±0.3	0.19	15.5
	5	1000±67	2216±313	31.8±0.4	6.8±0.33	2.9±0.2	0.24	16.1
	6	*	*	43.7±0.7	4.8±0.05	2.9±1.1	0.25	18.3
	7	1955±186	2133±300	74.3±0.9	3.0±0.02	2.4±0.3	0.22	14.4
	8	1886±138	3540±501	45.1±0.3	5.2±0.06	3.1±0.4	0.23	15.2
	9	1869±110	5405±764	86.4±7.8	3.6±0.31	2.6±2.3	0.24	15.7
$\mathrm{MDX}_{\mathrm{RAP-031}}$	1	1416±133	1595±225	41.5±0.9	4.3±0.05	2.9±0.8	0.25	16.3
	2	2076±206	2102±297	30.3±0.6	4.4±0.03	2.5±0.3	0.31	17.9
	3	1261±110	1658±234	50.8±1.2	4.4±0.05	2.4±0.1	0.33	16.3
	4	1921±187	2024±286	40.9±1.5	4.4±0.06	2.7±0.6	0.33	16.6
	5	1905±162	2623±371	39.1±0.4	4.4±0.02	4.1±0.3	0.33	16.7
	6	2449±169	5131±725	34.2±1.0	5.0±0.08	2.8±0.5	0.27	15.2
	7	1416±94	3189±451	55.9±1.2	4.0±0.05	3.0±0.5	0.31	16.7
	8	3525±185	12707±1797	42.8±0.5	5.0±0.04	2.8±0.2	0.31	17.9
	9	2318±146	5827±824	49.0±1.2	5.8±0.11	3.2±0.3	0.31	15.4
	10	1899±142	3367±476	41.8±0.7	4.2±0.05	3.2±0.1	0.24	15.1

* Not determined.

† Myofiber cross-sectional area distribution mean μ and standard deviation s parameters of a normal distribution $f(x;\mu,s) = \frac{1}{s\sqrt{2\pi}} e^{-\frac{(x-\mu)^2}{2s^2}}$.

‡ Myofiber cross-sectional area distribution scale μ and shape λ parameters of an inverse Gaussian distribution $f(x;\mu,\lambda) = \sqrt{\frac{\lambda}{2\pi x^3}} e^{\frac{-\lambda(x-\mu)^2}{2\mu^2 x}}$.

§ The estimated resistance ratio standard error is approximated as $\widehat{\sigma}_{\widehat{R}_0/\widehat{R}_\infty} \approx \sqrt{\left(\frac{1}{\widehat{R}_\infty}\right)^2 \widehat{\sigma}^2_{\widehat{R}_0} + \left(\frac{-\widehat{R}_0}{\widehat{R}^2_\infty}\right)^2 \widehat{\sigma}^2_{\widehat{R}_\infty}}$, assuming $\widehat{R}_0$ and $\widehat{R}_\infty$ uncorrelated variables.

⋆ The muscle mass (m) and the muscle optimal length (L_o) are not the result of an estimation process and therefore are denoted without $\widehat{\ }$.
